# Reduced occupancy of hedgehogs (*Erinaceus europaeus*) in rural England and Wales: The influence of habitat and an asymmetric intra-guild predator

**DOI:** 10.1038/s41598-018-30130-4

**Published:** 2018-09-06

**Authors:** Ben M. Williams, Philip J. Baker, Emily Thomas, Gavin Wilson, Johanna Judge, Richard W. Yarnell

**Affiliations:** 1 School of Biological Sciences, University of Reading, Whiteknights, Reading, Berkshire, RG6 6AS UK; 2 People’s Trust for Endangered Species, 3 Cloisters House, 8 Battersea Park Road, SW8 4BG London, UK; 3 Biocensus Limited, The Malt House, 17 – 20 Sydney Buildings, BA2 6BZ Bath, UK; 4 National Wildlife Management Centre, Animal and Plant Health Agency, Woodchester Park, GL10 3UJ Stonehouse, UK; 5 National Biodiversity Network, 12 - 14 St Mary’s Gate, Lace Market, NG1 1PF Nottingham, UK; 6 School of Animal, Rural and Environmental Sciences, Nottingham Trent University, Brackenhurst Campus, Nottinghamshire NG25 0QF Southwell, UK

## Abstract

Agricultural landscapes have become increasingly intensively managed resulting in population declines across a broad range of taxa, including insectivores such as the hedgehog (*Erinaceus europaeus*). Hedgehog declines have also been attributed to an increase in the abundance of badgers (*Meles meles*), an intra-guild predator. The status of hedgehogs across the rural landscape at large spatial scales is, however, unknown. In this study, we used footprint tracking tunnels to conduct the first national survey of rural hedgehog populations in England and Wales. Single and two-species occupancy modelling was used to quantify hedgehog occupancy in relation to habitat and predator covariates. Hedgehog occupancy was low (22% nationally), and significantly negatively related to badger sett density and positively related to the built environment. Hedgehogs were also absent from 71% of sites that had no badger setts, indicating that large areas of the rural landscape are not occupied by hedgehogs. Our results provide the first field based national survey of hedgehogs, providing a robust baseline for future monitoring. Furthermore, the combined effects of increasing badger abundance and intensive agriculture may have provided a perfect storm for hedgehogs in rural Britain, leading to worryingly low levels of occupancy over large spatial scales.

## Introduction

Quantifying the distribution and abundance of wildlife in relation to biotic, abiotic and temporal factors is fundamental to sound wildlife management^1^. The conservation status of the West European hedgehog (*Erinaceus europaeus*) throughout the United Kingdom is currently uncertain, although monitoring programmes based upon questionnaire surveys, timed observations in known habitats and counts of dead animals on roads indicate that numbers have declined markedly over the last two decades (e.g.^2–5^). In addition, a range of ecological and anthropogenic factors can be recognised which may have negatively impacted hedgehog populations.

Habitat loss is one of the main threats to global biodiversity and the key cause of species loss in terrestrial ecosystems^6–8^, and has been driven principally by the increased intensity of agricultural production^9–13^. Within the UK, agricultural landscapes have changed significantly since the early 1900s, becoming more intensively managed and homogenised through practices such as the removal of hedgerows to create larger fields^9,14^, the widespread application of molluscicides, insecticides and other pesticides^9,13^ and increased mechanisation. In the UK, one of the hedgehog’s preferred habitats, grassland, has declined in area since the 1950s^15^. Such changes have had detrimental impacts on a range of taxa^9,13,16,17^ and are likely to have negatively affected hedgehog populations by: reducing habitat heterogeneity^18^; affecting dispersal behaviour^19^; reducing invertebrate prey abundance^20^ and distribution^21,22^; and also possibly via the bioaccumulation of toxic compounds (e.g.^23^).

In addition, rural landscapes are further fragmented by road networks which could potentially act as a significant source of mortality and a barrier to movement^24,25^. For example, Rondinini and Doncaster^26^ identified that hedgehogs appeared to avoid crossing major roads, most likely as a response to the risk associated with crossing an increased number of lanes of traffic and/or the increased volume of traffic (but see^27^). Since 1970, the total length of motorways (the major road type in the UK) has increased from 1000 km to >3500 km^28^. Such avoidance and/or barrier effects could lead to the isolation of hedgehog populations, potentially making them more vulnerable.

Within the UK, hedgehogs have few natural predators^29^, but numbers of their principal predator, the Eurasian badger (*Meles meles*), have approximately doubled in the last 25 years following increased legal protection^30,31^. A range of studies in the UK^32–35^ and elsewhere^36^ have documented a negative relationship between hedgehog density/occupancy and badgers, although the mechanism behind this relationship is not fully understood. As an intra-guild predator of hedgehogs, badgers could potentially negatively affect hedgehog populations via direct predation and/or through increased competition for food resources; alternatively hedgehogs may preferentially occupy “refuge” habitats where badgers are rare or absent^36–38^. Historically, Micol *et al*.^39^ estimated that where badger main sett density exceeded 0.23 setts per km^2^, hedgehogs would be extirpated from all but isolated pockets; this main sett density has now been surpassed across much of England and Wales^30^.

The relative importance of the factors outlined above in affecting the current distribution and abundance of hedgehogs is, however, not known. This has, in part, been due to the absence of a reliable technique for surveying rural hedgehogs at the appropriate spatial scale^3^. For example, anthropogenic management practices are likely to vary within the rural landscape at the scale of individual properties such as farms and amenity sites, whereas approaches such as counts of road traffic casualties are typically conducted at much larger scales spanning multiple properties. Consequently, Yarnell *et al*.^35^ successfully developed and tested a survey method based upon the use of footprint tunnels to record the presence/absence of hedgehogs. In this study, we utilise that method to conduct the first national scale survey of rural hedgehog populations to: (i) measure levels of occupancy across rural England and Wales; and (ii) investigate relationships between habitat availability, predator abundance and patterns of occupancy. These data can then (iii) be used as a baseline against which future changes can be measured.

## Methods

**Figure 1:**
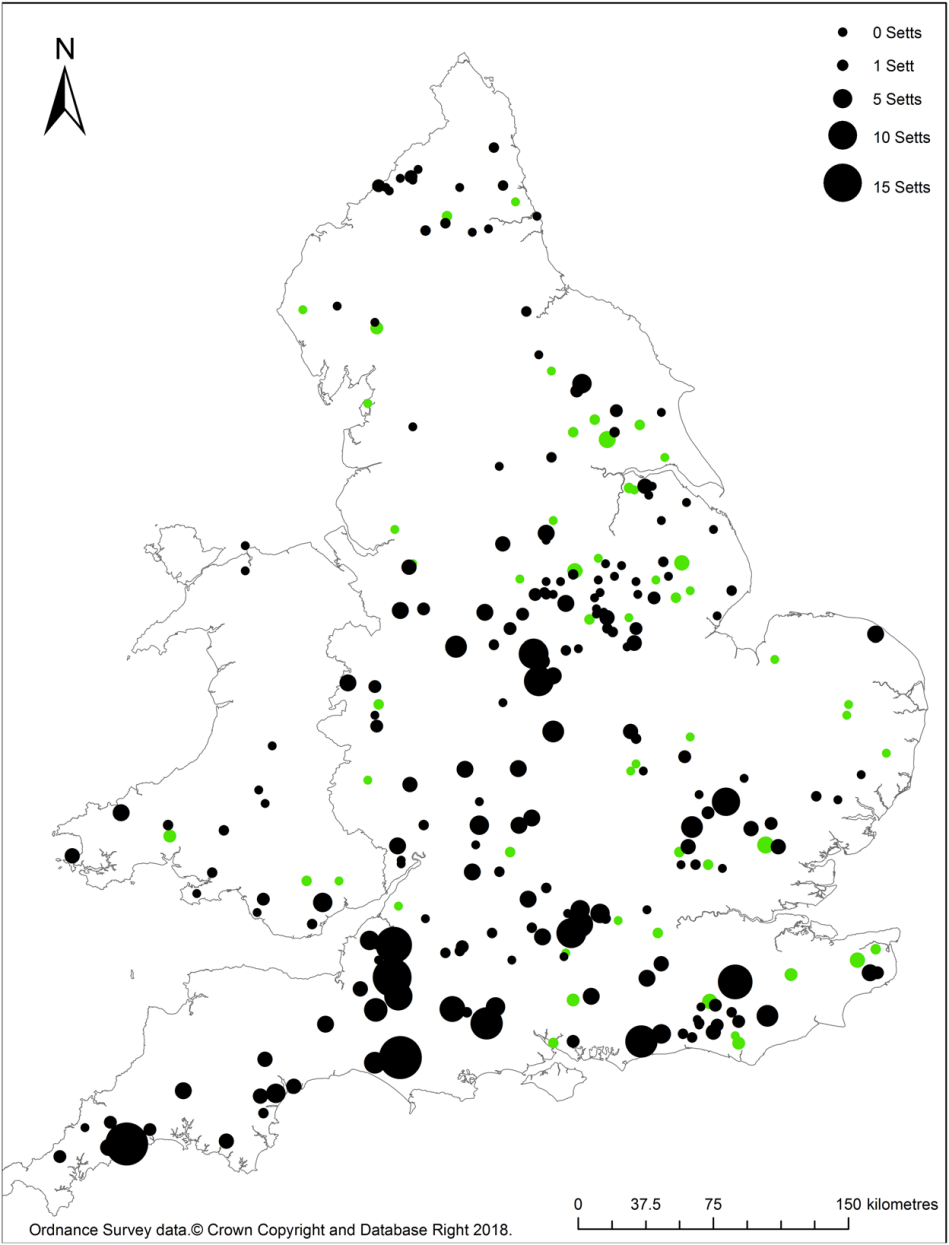
Pattern of hedgehog occupancy on sites surveyed in England and Wales in relation to relative badger density.  = hedgehog detected, ● = no hedgehog detected. The size of the circle indicates the number of badger setts at each site.

Sites (1 km Ordnance Survey grid squares) were surveyed between April-October inclusive in 2014–2015 (Fig. 1). Sites were selected randomly from 1 km squares surveyed as part of a prior national survey of badger setts in November 2011-March 2013^30^, stratified by land class^40^. As the focus of both surveys was on rural populations, squares had been excluded if they contained >50% urban area.

Surveys were conducted by volunteers and university students supervised by the authors. Surveyors were asked to survey an area of approximately 500 m × 500 m near the centre of their allocated square(s) and which was owned/managed by one person or organisation. Volunteers were provided with all field equipment and a comprehensive manual detailing the background to the project, survey methodology, health and safety information, data recording sheets and example hedgehog footprint sheets. No prior knowledge of hedgehog status at any site was known and all knowledge of badger activity at sites was withheld from surveyors.

Ten footprint tunnels were deployed at each site placed parallel to linear features (e.g. woodland edges, hedgerows, fences) as hedgehogs frequently follow these when travelling^41^. Tunnels were placed >100 m apart, with no more than two tunnels in the same field^35^. Tunnels were checked daily for five continuous days: food bait (commercially available dry hedgehog food) was replaced if necessary and footprint papers were replaced if they were damaged or had recorded hedgehog or non-target animal footprints. All footprint papers were returned for verification by the authors.

### Factors affecting hedgehog occupancy

Single-species single-season occupancy models were first used to examine hedgehog presence/absence in relation to habitat availability, habitat complexity and relative badger density. Occupancy models use repeated detection/non-detection data of a species over a series of surveys to estimate its occurrence and relationship with covariates whilst allowing for imperfect detection^42^. Each survey night was treated as a repeat survey; tunnels were not considered independent as individual hedgehogs could have visited >1 tunnel each night. Sites were therefore classified as occupied if ≥1 tunnel recorded hedgehog footprints on any night. Data were analysed after pooling across both years. Naïve occupancy is defined as the proportion of sites surveyed where hedgehogs were detected; true occupancy is the proportion of sites estimated to be occupied after taking the false-absence error rate into account. All occupancy analyses were conducted using PRESENCE v12.6^43^.

Habitat variables that were expected to directly or indirectly influence hedgehog occupancy and/or detection at different scales were included in occupancy models. Larger-scale effects were investigated by incorporating individual and merged land classes (1 km^2^ resolution^40^; Tables 1 and 2); these represent broad habitat types and general patterns of land use within survey squares. Finer-scale effects were investigated using the aerial availability of four land cover types aggregated from UK Biodiversity Action Plan Broad Habitats as hedgehog abundance is known to vary markedly between habitats (e.g^36,39,44^): BUILT (built, urban and suburban habitats combined); WOODLAND (broadleaved and coniferous woodland combined); GRASSLAND (all grassland habitats combined); and ARABLE. Habitat availability (25 m^2^ resolution from Landcover 2007 maps^45^) was calculated as the proportion of the 1 km grid square area; data were arcsine square root transformed for analysis.

**Table 1:** Summary of the covariates used in the single-season single-species occupancy models^42^ and data format for each. Land classes are described in Table 2.

Variable name	Description	Variable type
LANDCLASS	All seven land classes	Binary for each land class
LCARABLE	Land classes 1, 2 and 3 merged	Binary
LCPASTORAL	Land classes 4 and 5 merged	Binary
LCUPLANDS	Land classes 6 and 7 merged	Binary
ARABLE	Proportional area of arable habitat in the survey square	Arcsine square root transformed
GRASSLAND	Proportional area of grassland habitat in the survey square	Arcsine square root transformed
BUILT	Proportional area of built habitat in the survey square	Arcsine square root transformed
WOODLAND	Proportional area of woodland habitat in the survey square	Arcsine square root transformed
SETTS	Number of badger setts in the survey square	Z-scores
ALLROADS	Total length (km) of roads in the survey square	Z-scores
MOTORWAY	Length (km) of motorways in the survey square	Z-scores
AROADS	Length (km) of dual carriageways and ‘A’ roads in the survey square	Z-scores
BROADS	Length (km) of ‘B’ roads in the survey square	Z-scores
MINORROADS	Length (km) of all minor (e.g. residential) roads in the survey square	Z-scores
HABITATS	Number of different habitat types in the survey square	Z-scores

**Table 2:** Descriptions of the seven land class groups used (from^30^) in the current study, and a summary of the number of sites surveyed, the number of sites where hedgehogs were detected (naïve occupancy), the number of sites where badger setts were detected and relative badger sett density.

Land class	Subclass	Description	% area of England and Wales	No. (%) of sites surveyed	No. (%) of sites where hedgehogs were detected	No. (%) of sites where badger setts were detected	Mean ( ± SD) badger sett density
Arable	1	Open, gentle slopes, varied agriculture, often wooded or built-up	9.6%	33 (13%)	4 (12.1%)	28 (84.8%)	3.36 ± 3.64
	2	Flat, arable and intensive agriculture, often cereals & grass mixtures	31.7%	106 (41%)	28 (26.4%)	60 (56.6%)	1.52 ± 2.15
	3	Lowlands with variable land use, mainly arable and intensive agriculture	2.3%	8 (3%)	2 (25.0%)	2 (25.0%)	0.25 ± 0.46
Pastoral	4	Undulating country, gently rolling enclosed country mainly fertile pastures. Some coastal areas mainly pasture with varied morphology and vegetation.	21.0%	58 (22%)	7 (12.1%)	43 (74.1%)	3.02 ± 3.59
	5	Heterogeneous land-use, includes flat plains, valley bottoms and undulating lowlands with mixed agriculture including pastoral and arable	17.8%	37 (14%)	10 (27.0%)	24 (64.9%)	1.57 ± 2.02
Marginal upland		Rounded hills and slopes, wide range of vegetation types including moorland and improvable permanent pasture	14.7%	12 (5%)	4 (33.3%)	5 (41.7%)	1.25 ± 1.90
Upland		Mountainous, with moorlands, afforestation and bogs	3.0%	7 (3%)	0 (0.0%)	2 (28.6%)	0.43 ± 0.79

In addition, as hedgehogs have been shown to avoid crossing major roads^26^, and hedgehog presence may be influenced by road density^46^, we incorporated five measures of road “availability”. These were the total length of: (i) all roads in the survey square (ALLROADS); (ii) motorways (MOTORWAY: in rural areas in the UK these typically have 6 lanes of traffic, a speed limit of 70 mph and a central median); (iii) “A” roads and dual carriageways (AROADS: these typically have 2 or 4 lanes of traffic, with a speed limit of 60 or 70 mph; dual carriageways also have a central median); (iv) “B” roads (BROADS: these are typically single lane roads with no central median and a speed limit of 40–60 mph); and (v) minor roads (MINORROADS: typically these are associated with villages and built up areas with a speed limit of 30 mph). Lengths were determined from the OS Meridian™ 2 data set in ArcMap 10.1; data were converted to z-scores for analysis as recommended by^47^.

Habit complexity (HABITATS) was defined as the number of different habitat types excluding roads (maximum = 23) in the survey square. Data were obtained from Landcover 2007 maps^45^.

The number of badger setts (main, subsidiary, annex and outlier) in each survey square (SETTS) was used as a measure of relative badger abundance^30^. Badger surveys were conducted by trained surveyors employed by the National Wildlife Management Centre^30^. Sites were surveyed on foot looking for refugia (setts). Both sides of all field boundaries were surveyed, and any badger runs radiating from boundaries into the middle of fields were followed if there was a possibility they would lead to a badger sett (e.g. to a small copse). Woodland and other rough terrain was surveyed using transects; particularly difficult terrain was walked by teams of surveyors walking parallel transects in visual contact with one another.

As sample sizes were moderate, individual occupancy models included a maximum of two covariates for occupancy and one for detection. In addition, preliminary analyses of potential associations between the number of badger setts (SETTS) and aerial habitat availability indicated a significant correlation with the area of GRASSLAND (Pearson’s Correlation Coefficient, r = 0.164, df = 260, P = 0.008) but not any other habitat type. Consequently, these two variables were not modelled together as explanatory covariates of occupancy or detection, but both were permitted in models using each for either occupancy or detection (i.e. a model including both GRASSLAND plus SETTS for occupancy was excluded, but a model with SETT as a covariate for occupancy and GRASSLAND as a covariate for detection was included).

The goodness of fit for the most global model was assessed using a bootstrap method (1000 replications) resulting in a variance inflation factor of ĉ = 1.67, and standard errors were inflated by a factor of $\sqrt{\hat{{\rm{c}}}}=1.29$. As data were over-dispersed, adjustments were made to the variance inflation factor (ĉ) and models were ranked by quasi-AIC (ΔQAIC) values^48^. Models with ΔQAIC values > 2 were regarded as having little or no support^48^. Models that did not converge were excluded.

### Further investigation of the relationship with badgers

As the number of badger setts was significantly related to hedgehog occupancy (see Results), a two-species occupancy model was used to estimate a Species Interaction Factor (SIF) between hedgehog and badger occupancy^42,49^; this is a ratio of the likelihood of the two species co-occurring compared to a hypothesis of independence. A value < 1 indicates avoidance (i.e. the two species co-occur less frequently than would be expected if they were distributed independently) whereas a value > 1 indicates aggregation (i.e. the two species co-occur more frequently than would be expected if they were distributed independently; e.g.^50,51^). As two-species occupancy models tend not to converge when covariates are added, they were omitted^52^.

To investigate whether the relationship between badger sett density and hedgehog presence/absence has changed since that reported by Micol *et al*.^39^, a polynomial regression analysis was used to estimate the density at which naïve hedgehog occupancy would be zero. Regression analysis was performed in Minitab 16.1.1. All figures are mean (±SE) unless otherwise stated.

### Data availability

The datasets generated and/or analysed during the current study are available from the corresponding author on reasonable request.

## Results

### Site characteristics

Overall, 261 sites were surveyed; 83 in 2014 and 178 in 2015. Eighteen sites were surveyed in Wales and 243 in England (Fig. 1) covering all seven land class groups (Table 2). Badger setts were found at 163 (62%) sites. The number of badger setts per survey square ranged from 0–16 (mean: 2.0 ± 0.2 setts km^−2^); the number of habitats present at each site ranged from 0–11 (5.2 ± 0.1). The most commonly occurring habitat type was GRASSLAND (253 sites; 97%), followed by ARABLE (243 sites; 93%), WOODLAND (219 sites; 84%) and BUILT (148 sites; 57%). On average, ARABLE, GRASSLAND, WOODLAND and BUILT habitats covered 45% ± 2%, 36% ± 2%, 11% ± 1% and 5% ± 1% of each survey square, respectively. The total length of roads per survey square ranged from 0.00–6.78 km (mean: 1.71 ± 0.09 km): the majority of roads were classified as minor (MINORROADS: 1.34 ± 0.07 km), followed by A-roads (AROADS: 0.20 ± 0.03 km) and, B-roads (BROADS:0.14 ± 0.02 km); MOTORWAY accounted for the lowest density (0.02 ± 0.01 km).

No badger setts or hedgehogs were detected at 70 (27%) sites; badger setts were detected at 163 (62%) sites and hedgehogs at 55 (21%) sites. Badger setts and hedgehogs were both found at 27 (10%) sites with only badger setts or hedgehogs being detected at 136 (52%) and 28 (11%) sites respectively.

### Patterns of occupancy

Hedgehogs were detected in only 55 sites, indicating an overall naïve occupancy rate of 21.1%. Within land classes, naïve occupancy rates varied from 0.0% to 33.3%, although sample sizes were small in some categories; for those land classes where >30 sites were surveyed, naïve occupancy rates varied from 12.1% to 27.0% (Table 2). Comparable figures for merged land class groupings were: arable 23.1% (N = 147); pastoral 17.9% (N = 95); and uplands 21.1% (N = 19). Accounting for the area of each land class in England and Wales, this gives an overall occupancy rate across England and Wales of 22.3%.

**Figure 2:**
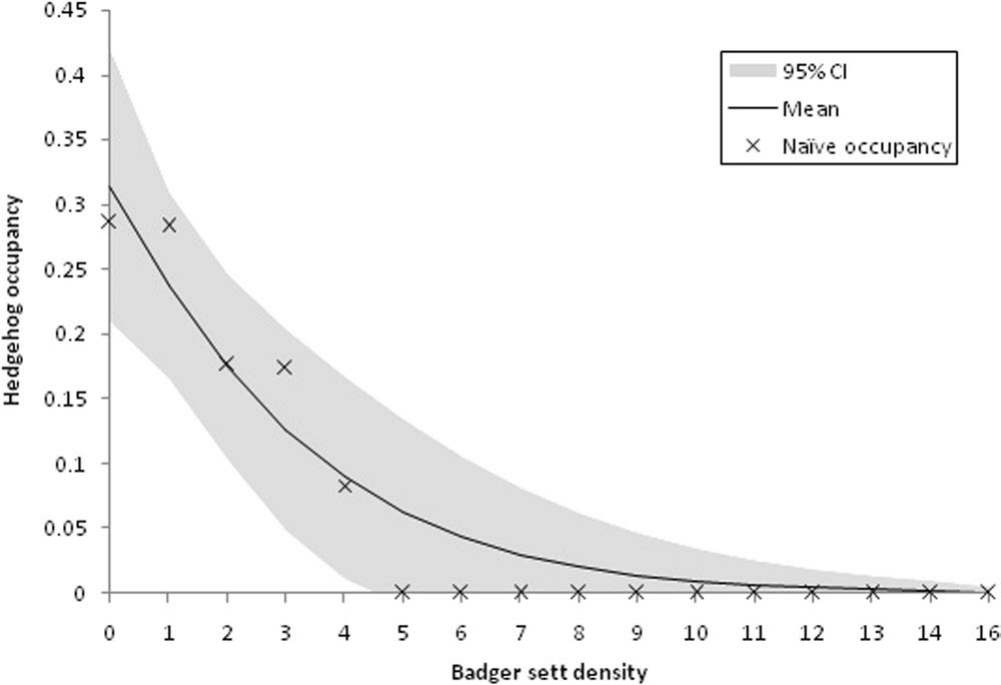
Relationship between total badger sett density (SETTS km^-2^) and hedgehog occupancy in England and Wales 2014–15. Black line indicates the mean number of sites occupied; shaded area indicates 95% confidence interval; naïve occupancy rates are indicated by x. The probability of hedgehog occupancy was based on an occupancy model with sett density added as a covariate, and constant detection.

**Figure 3:**
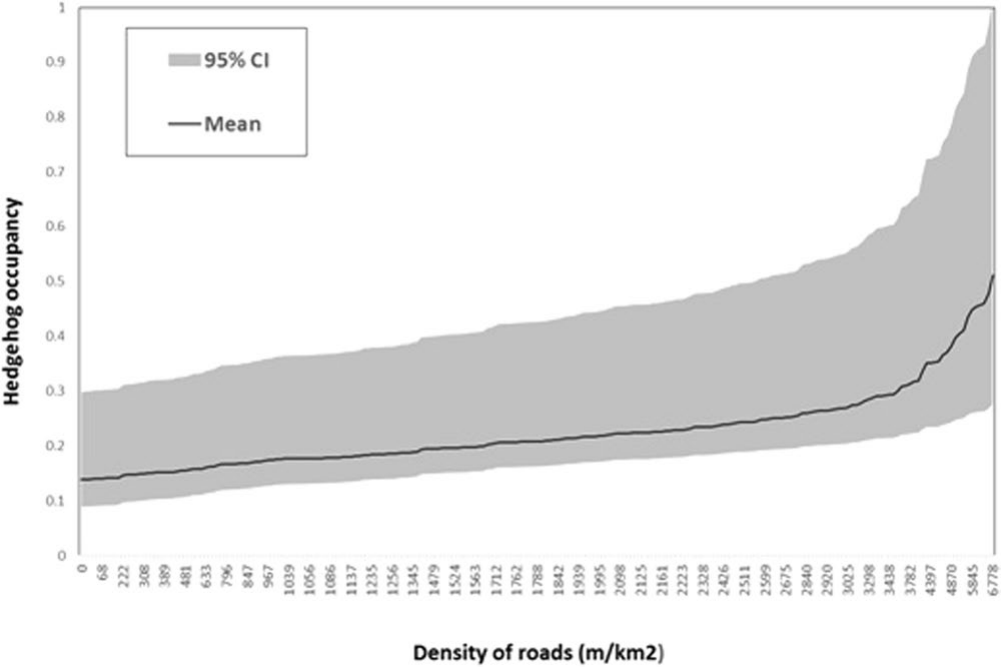
Relationship between road density (m/km^2^) and hedgehog occupancy in England and Wales in 2014–15. Probability of hedgehog occupancy was based on an occupancy model with the length of all roads added as a covariate, and constant detection.

The best fitting models for hedgehog occupancy included relative badger abundance (SETTS) and two measures of urbanisation (BUILT and ALLROADS), with detection influenced by the proportional area of GRASSLAND and the number of different habitats (HABITATS) (Table 3). These covariates made up the five best fitting models, with a combined QAIC weight of 0.61; all five models contained SETTS as a covariate of occupancy. In the highest ranked model, relative badger abundance was significantly negatively associated with hedgehog occupancy (β = −1.14, 95% CI = −0.3,−1.97) (Fig. 2), whereas the total length of all roads had a significant positive relationship (β = 0.41, 95% CI = 0.04, 0.78) (Fig. 3). There was also some support for hedgehog occupancy being positively related to the proportion of BUILT area at a site (β = 1.90, 95% CI = −0.04, 3.84) in the third highest ranked model, although this was not significant. GRASSLAND was positively associated with hedgehog detection (β = 1.25, 95% CI = −0.09, 2.59) in the top ranked model, and a negative relationship with number of habitats also gained support in two of the top 5 ranked models (β = −0.32, 95% CI = −0.70, 0.05), although these were not significant.

**Table 3:** Summary of single-species occupancy models run on the complete data set (N = 261 sites). The top ranked models (ΔQAIC < 2.0) are in bold. Variables are listed in Table 1. Ψ: occupancy; P: detection.

Model	QAIC	∆QAIC	AICwgt	Model likelihood	No. of parameters
**Ψ(SETTS + ALLROADS),P(GRASSLAND)**	364.04	0.00	0.182	1.0000	5
**Ψ(SETTS + ALLROADS),P(HABITATS**)	364.71	0.67	0.130	0.7153	5
**Ψ(SETTS + BUILT),P(GRASSLAND)**	364.84	0.80	0.122	0.6703	5
**Ψ(SETTS + ALLROADS),P(.)**	365.46	1.42	0.089	0.4916	4
**Ψ(SETTS + BUILT),P(HABITATS)**	365.49	1.45	0.088	0.4843	5
Ψ(SETTS + BUILT),P(.)	366.26	2.22	0.060	0.3296	4
Ψ(SETTS),P(GRASSLAND)	366.46	2.42	0.054	0.2982	4
Ψ(SETTS),P(HABITATS)	367.21	3.17	0.037	0.2049	4
Ψ(SETTS),P(AROADS)	367.8	3.76	0.028	0.1526	4
Ψ(SETTS + WOODLAND),P(GRASSLAND)	367.92	3.88	0.026	0.1437	5
Ψ(SETTS),P(.)	367.92	3.88	0.026	0.1437	3
Ψ(SETTS),P(ARABLE)	368.47	4.43	0.020	0.1092	4
Ψ(SETTS),P(LCUPLANDS)	368.58	4.54	0.019	0.1033	4
Ψ(SETTS),P(BUILT)	368.59	4.55	0.019	0.1028	4
Ψ(SETTS),P(MOTORWAY)	369.09	5.05	0.015	0.0801	4
Ψ(SETTS),P(LCPASTORAL)	369.29	5.25	0.013	0.0724	4
Ψ(SETTS),P(BROADS)	369.44	5.40	0.012	0.0672	4
Ψ(SETTS),P(WOODLAND)	369.67	5.63	0.011	0.0599	4
Ψ(SETTS),P(SETTS)	369.85	5.81	0.010	0.0547	4
Ψ(SETTS),P(ALLROADS)	369.87	5.83	0.010	0.0542	4
Ψ(SETTS),P(MINORROADS)	369.89	5.85	0.010	0.0537	4
Ψ(SETTS),P(LCARABLE)	369.89	5.85	0.010	0.0537	4
Ψ(BUILT),P(GRASSLAND)	373.25	9.21	0.002	0.0100	4
Ψ(ALLROADS),P(GRASSLAND)	373.25	9.21	0.002	0.0100	4
Ψ(ALLROADS),P(.)	374.61	10.57	0.001	0.0051	3
Ψ(BUILT),P(.)	374.62	10.58	0.001	0.0050	3
Ψ(MINORROADS),P(GRASSLAND)	374.65	10.61	0.001	0.0050	4
Ψ(BROADS),P(GRASSLAND)	374.67	10.63	0.001	0.0049	4
Ψ(.),P(GRASSLAND)	375.53	11.49	0.001	0.0032	3
Ψ(WOODLAND),P(GRASSLAND)	376.01	11.97	0.001	0.0025	4
Ψ(SETTS),P(LANDCLASS)	376.26	12.22	0.000	0.0022	10
Ψ(.),P(.)	376.9	12.86	0.000	0.0016	2
Ψ(LCPASTORAL),P(GRASSLAND)	376.95	12.91	0.000	0.0016	4
Ψ(LCARABLE),P(GRASSLAND)	376.99	12.95	0.000	0.0015	4
Ψ(ARABLE),P(GRASSLAND)	377.2	13.16	0.000	0.0014	4
Ψ(AROADS),P(GRASSLAND)	377.28	13.24	0.000	0.0013	4
Ψ(HABITATS),P(GRASSLAND)	377.37	13.33	0.000	0.0013	4
Ψ(MOTORWAY),P(GRASSLAND)	377.44	13.40	0.000	0.0012	4
Ψ(LCUPLANDS),P(GRASSLAND)	377.53	13.49	0.000	0.0012	4
Ψ(GRASSLAND),P(.)	378.35	14.31	0.000	0.0008	3
Ψ(HABITATS),P(.)	378.74	14.70	0.000	0.0006	3
Ψ(.),P(*variable detection*)	381.84	17.80	0.000	0.0001	6
Ψ(LANDCLASS),P(GRASSLAND)	382.43	18.39	0.000	0.0001	10

### Further investigation of the relationship with badgers

The two-species occupancy models showed the probability (mean ± SE) that hedgehogs would be present at a site: (a) regardless of the presence of badger setts was 21.1% ± 3.2%;(b) given that badger setts were present was 17.8% ± 3.6%; and (c) given that badger setts were not present was 31.0% ± 7.0%. The probability of detecting hedgehogs rose from 59.3% ± 6.2% when no badger setts were present to 62.2% ± 4.1% when badger setts were present. The Species Interaction Factor was 0.670 ± 0.126, indicating that hedgehogs were significantly less likely to co-occur with badgers than would be expected under an independence hypothesis (i.e. hedgehogs show avoidance of badgers; 95% CI: 0.503, 0.891).

**Figure 4:**
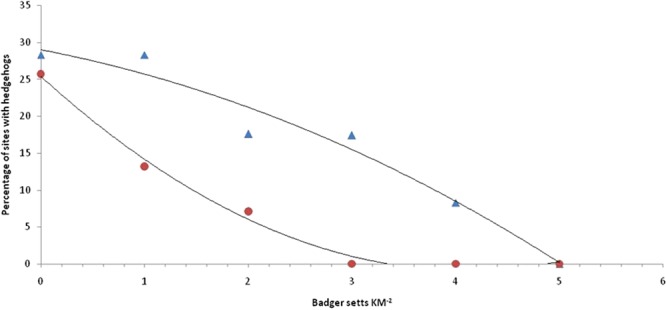
Relationship between hedgehog occupancy and density of badger setts. The percentage of sites where hedgehogs were detected regressed against density of all badger setts (▲F_1,4_ = 60.12, P < 0.001; y = −0.626x^2^ − 2.628x + 28.96, R² = 0.961) and main setts only (●F_1,3_ = 28.06, P = 0.01; y = 1.551x^2^ − 12.76x + 25.35, R² = 0.992).

The predicted sett density above which the probability of a site being occupied by hedgehogs becomes zero was 5.21 setts km^−2^ (95% CI: 4.07, 6.35) or 3.29 main setts km^−2^ (95% CI: 2.17, 4.40 (Fig. 4).

## Discussion

Agricultural landscapes have become more intensively managed and homogenised resulting in population declines across a range of taxa^13,16^. In the UK, the hedgehog, a generalist insectivore, may be one such species^3,5^. Hedgehogs select habitats with high prey availability^38^, and which provide secure resting, breeding and hibernating sites safe from predators^41^. However, the current rural landscape is often lacking such habitats (e.g.^9,14,15^), leading some to suggest that the wider British landscape has become “unsuitable” for hedgehog populations^37^. Unfortunately, however, there is a lack of empirical data regarding historical hedgehog abundance and distribution at the landscape scale, making inferences on the magnitude of population change difficult.

One reason for this lack of information has been the practical problems associated with surveying hedgehog populations within rural habitats^35^. Although hedgehog populations have been relatively well studied in urban areas (e.g.^4,53–56^), studies in rural landscapes have typically been conducted either at a local level (e.g.^22,37,38,57,58^), such that their results may not be representative of larger geographic scales, or at a large-spatial scale that makes it difficult to clearly identify underlying biological and/or anthropogenic influences (e.g.^59^). Therefore, the data presented in the current study represent the first national scale estimate of hedgehog occupancy across rural England and Wales; as such, these can be used as a baseline against which any future changes can be measured.

Hedgehogs were widely distributed across England and Wales, being found in all but one land class (upland): this is not surprising as hedgehogs are known to be absent above the tree line^29^. Occupancy rates were, however, low across all other land classes as well, ranging from 12–33%. We contend that this is the first unbiased estimate of occupancy at a landscape scale, since the selection of study sites was random, with each land class being surveyed in proportion to its coverage. Consequently, it is to be expected that this study would provide a lower estimate of occupancy (22.3% across all land classes) compared to Yarnell *et al*.^35^ (39.2%) who used exactly the same methodology, but where sites were biased towards pasture and amenity grasslands situated close to urban areas, which hedgehogs seem to prefer^39^.

The occupancy estimate recorded here is also lower than in other studies conducted at smaller scales and using different methods: 26% of amenity grasslands in villages^33^; 36–45% of gardens in urban areas, 30–55% of farms and 47–57% of roads^3,56,60,61^. This apparently patchy distribution of rural hedgehog populations may suggest that some populations are isolated and fragmented^24^. Consequently, there is an urgent need to investigate patterns of gene flow between populations of hedgehogs in relation to potential physical obstacles such as major roads, but also in relation to less visible biological obstacles such as predator/competitor populations (see below).

We were not able to detect any significant influence of the aerial availability of rural habitat types on hedgehog occupancy, nor any effect of habitat complexity, although this may have, in part, been constrained by the low number of sites where hedgehogs were recorded. Our study did, however, detect a positive relationship between hedgehogs and both the proportional area of built habitat and total road density. This is consistent with previous radio-tracking studies that have demonstrated that hedgehogs prefer to occupy areas associated with human habitation rather than the wider countryside, as these may be associated with e.g. reduced badger abundance, increased food availability and/or novel refugia^32,36,38,41,62^. Similarly,^46^ found a significant positive correlation between hedgehog presence/absence and road density in the Netherlands (but see^25^); this is likely, in part, to reflect a similar association with areas of human habitation, as road density will increase with increasing housing coverage.

The major explanatory variable in our occupancy models, however, was relative badger density, quantified as the total number of all sett types present in survey squares^30^. We elected to use this variable rather than the number of main setts alone (which is typically used to estimate the number of badger social groups^30,31^) as it is arguably likely to better represent the intensity of use of the survey site by badgers (e.g. where main setts were not present within the 1 km survey square itself, the site itself is still likely to be used by neighbouring groups).

Badgers are the main predator of hedgehogs but also competitors for food resources, and an increasing number of studies have shown a negative association between the two species in terms of density^34^, occupancy^35^, and also spatial separation at the local scale (e.g.^32,36,38,41,61^) although Haigh *et al*.^57^ reported co-occurrence locally in Ireland. The two different occupancy models (i.e. single-species and two-species) presented here support these studies, showing a negative relationship between badger sett density and hedgehog occupancy. However, this relationship appears complex.

For example, of those 55 sites where hedgehogs were present, badgers were also present on 49.1% of these sites. This demonstrates that badgers and hedgehogs can, and do, coexist at the 1 km^2^ scale^57^. The extent to which the ranging patterns of the two species overlap in space and/or time is, however, not known, although this does not appear to be a simple case of hedgehogs “hiding” in built environments, as footprint tunnels were placed in rural habitats. Consequently, there is the need for studies focussed on the behaviour of sympatric hedgehogs and badgers to investigate how the two species can live alongside one another, and what factors promote this co-existence.

However, the probability of hedgehog occupancy did decline as the number of badger setts increased: naïve occupancy was 28.6% where badger setts were not present, but only 16.6% where they were present. As outlined above, it is plausible that an increase in sett numbers does reflect an increased level of badger activity/intensity, although the continually changing and highly variable nature of badger social groups and densities makes it impossible to directly relate sett density to badger density (e.g.^63–65^). Despite these caveats, the sett density predicted here where hedgehogs would no longer occupy an area (5.21 setts km^−2^ or 3.29 main setts/km^−2^) is far greater than that reported by Micol *et al*.^39^ (≥0.23 main setts km^−2^). This will in part be down to methodological differences as Micol *et al*.’s^39^ prediction was based on hedgehog abundance whereas we have been limited to hedgehog presence/absence. Micol *et al*.^39^ also acknowledged that hedgehogs would still be present in isolated areas whereas our prediction is for complete extirpation.

In the context of the current distribution and abundance of badgers in the UK following their increased legal protection since 1992, the threshold density estimated by Micol *et al*.^39^ has already been surpassed for much of England and Wales (the exception is Land Class 7^30^). This raises significant concerns for the future of hedgehogs in rural environments in the UK, although we predict that badger main sett density would have to increase more than six fold from that reported by Judge *et al*.^30^ for badgers to completely extirpate hedgehogs from England and Wales: for comparison, the density of main setts increased by approximately 24% between 1988 and 1997^66^, and by 88% between 1988 and 2013^30^. Furthermore, given the absence of information concerning the biological mechanism(s) by which this negative association arises, it is reasonable to suppose that changes in badger numbers alone might not necessarily be the only factor affecting future changes in hedgehog populations. For example, hedgehogs may be able to persist in areas not used extensively by badgers, as predicted by intra-guild predation theory^67^.

Whilst badgers are clearly negatively associated with hedgehog occupancy, over a quarter (26.8%) of the sites surveyed in this study had no badger setts or hedgehogs present; in addition, the two-species occupancy modelling estimated that the probability that hedgehogs would be present at a site given that no badger setts were present was still only 31.0%. These figures would seem to indicate that a large proportion of rural England and Wales is unsuitable for both species. Given the similarity in diets of the two species^44,68^, one plausible explanation for this result might be the reduced availability of macro-invertebrate prey in relation to factors such as agricultural intensification and climate change. In addition, this might also suggest that hedgehog occupancy would still be worryingly low even if badger numbers were reduced, for example during culling programs designed to reduce the incidence of bovine tuberculosis in cattle^69^.

In summary, much of the blame for the perceived hedgehog decline in the UK has focussed upon the impacts of badgers as both a competitor but especially as a predator (e.g.^34^). Although our findings support the negative relationship between the two species, this relationship is likely to be complex, involving elements of predation, competition and avoidance; in the context of the latter, areas associated with human habitation appear to mitigate against some of the negative effects of badgers. At the same time, however, rates of hedgehog occupancy were low even in the absence of badgers, and badger setts were not recorded in 47.9% of sites surveyed. Collectively, this suggests that intensive management of rural areas is negatively impacting both these generalist terrestrial insectivores. Future work must, therefore, focus on identifying the exact biological mechanism(s) by which badgers negatively impact hedgehogs, and how these impacts can be managed effectively to promote the co-existence of these species.
